# Licorice Ameliorates Cisplatin-Induced Hepatotoxicity Through Antiapoptosis, Antioxidative Stress, Anti-Inflammation, and Acceleration of Metabolism

**DOI:** 10.3389/fphar.2020.563750

**Published:** 2020-11-10

**Authors:** Qiong Man, Yi Deng, Pengjie Li, Jun Ma, Zhijun Yang, Xiujuan Yang, Yan Zhou, Xiao Yan

**Affiliations:** ^1^ College of Pharmacy, Gansu University of Chinese Medicine, Lanzhou, China; ^2^ School of Pharmacy, Chengdu Medical College, Chengdu, China; ^3^ Key Laboratory of Pharmacology and Toxicology of Traditional Chinese Medicine of Gansu Province, Lanzhou, China; ^4^ Department of Pharmacy, The First Hospital of Lanzhou University, Lanzhou, China

**Keywords:** cisplatin, detoxification, hepatotoxicity, licorice, proteomics

## Abstract

Cisplatin (CP) is one of the most effective antitumor drugs in the clinic, but has serious adverse reactions, and its hepatotoxicity has not been fully investigated. Licorice (GC), a traditional herbal medicine, has been commonly used as a detoxifier for poisons and drugs, and may be an effective drug for CP-induced hepatotoxicity. However, its mechanism and the effector molecules remain ambiguous. Therefore, in this study, a network pharmacology and proteomics-based approach was established, and a panoramic view of the detoxification of GC on CP-induced hepatotoxicity was provided. The experimental results indicated that GC can recover functional indices and pathological liver injury, inhibit hepatocyte apoptosis, upregulate B-cell lymphoma/leukemia 2 (Bcl-2) and superoxide dismutase (SOD) levels, and downregulate cellular tumor antigen p53 (p53), caspase-3, malondialdehyde high mobility group protein B1 (HMGB1), tumor necrosis factor alpha (TNF-α), and interleukin 1β (IL-1β) levels. Proteomics indicated that GC regulates phosphatidylcholine translocator ABCB1 (ABCB1B), canalicular multispecific organic anion transporter 1 (ABCC2), cytochrome P450 4A2 (CYP4A2), cytochrome P450 1A1 (CYP1A1), cytochrome P450 1A2 (CYP1A2), estrogen receptor (ESR1), and DNA topoisomerase 2-alpha (TOP2A), inhibits oxidative stress, apoptosis, and inflammatory responses, and accelerates drug metabolism. In this study, we provide the investigation of the efficacy of GC against CP-induced hepatotoxicity, and offer a promising alternative for the clinic.

## Introduction

Cisplatin (CP) has been widely used in ovarian cancer, prostate cancer, testicular cancer, and others, due to its potent penetration and wide anticancer spectrum (Dasari and Tchounwou, 2014). However, severe ototoxicity, nephrotoxicity, and hepatotoxicity activities limit its use in the clinic (Barabas et al., 2008). In several studies, it was indicated that long-term accumulation of large doses of CP can cause hepatotoxicity by inducing oxidative stress, inflammatory, and hepatocellular apoptosis (Omar et al., 2016; Qi et al., 2019). In fact, the accumulation of CP in the liver is second only to that in the kidney (Bandu et al., 2015), whereas limited attention has been paid to hepatotoxicity, and the underlying mechanism, as well as protective drugs, has not been thoroughly studied. Licorice (Chinese name GanCao, GC, the roots of *Glycyrrhiza uralensis* Fisch. ex DC.) is widely used for its antibacterial, anti-inflammatory, antioxidant, and anticancer properties. GC and its active components, including glycyrrhizin, glycyrrhetinic acid, and glycyrrhetinic, have remarkable detoxification effects on drugs and poisons by relieving the toxic symptoms and reducing the death rate (Pastorino et al., 2018; He et al., 2019). Moreover, GC possesses significant hepatoprotective activities to liver injury by CCl_4_ or acetaminophen (Kuang et al., 2017; Wu et al., 2020). Some antioxidant enzymes, such as superoxide dismutase (SOD), glutathione S-transferase (GST), glutathione peroxidase (GPx), can be induced by GC (Hejazi et al., 2017). Although it has previously been reported that functional indices in CP-induced liver injury recovered by administration of GC (Lee et al., 2007), functional characteristics and underlying mechanism have not yet been elucidated.

Due to synergistic effects by complex components of GC, it remains a challenge to determine the molecular targets and the mechanism involving detoxification of GC on CP-hepatotoxicity. To solve this problem, a molecular network with GC-effective compounds, targets, and CP toxic targets can be established by network pharmacology. Moreover, changes in protein expression in toxic organs can be analyzed by proteome to explain the biological process involved (Suman et al., 2016), which is also helpful in elucidating the molecular mechanism of action. Thus, in this study, we established a CP-induced hepatotoxicity model in rats, observed the detoxification of GC, and discussed the mechanisms involved based on potential intervention targets by network pharmacology and proteomic analysis. Taken together, this study provides a panoramic basis for the rational clinical application of GC and CP.

## Materials and Methods

### Materials and Reagents

CP of over 98.5% purity (by HPLC) was obtained from Beijing Solarbio Technology Co., Ltd. (Beijing, China). Wild roots of *Glycyrrhiza uralensis* Fisch. ex DC. were obtained from Jinta, Jiuquan (40°06′47″N 99°24′01″E, Gansu, China), and authenticated by Prof. Ling Jin (Gansu University of Chinese Medicine). Voucher specimens were deposited in the Institute of Medicinal Plant Development, Chinese Academy of Medical Sciences (NYBG Herbarium Code: IMD). 100 g wild GC powder was extracted with 1,000 ml of 70% ethanol that was refluxed for 3 h twice, filtered, combined the filtrate, concentrated to water evaporation for reserve (Zhu et al., 2018), and then, lyophilized into powders (46.3% yield). Antibodies directed against phosphatidylcholine translocator ABCB1 (ABCB1B), canalicular multispecific organic anion transporter 1 (ABCC2), cytochrome P450 1A1 (CYP1A1), cytochrome P450 1A2 (CYP1A2), estrogen receptor 1 (ESR1), and DNA topoisomerase 2-alpha (TOP2A) were purchased from Abcam Biotechnology Co. (Milton, Cambridge, UK). Cytochrome P450 4A2/1 (CYP4A2) antibody was purchased from Affinity Biosciences (Beijing, China). All other chemicals were reagents of analytical-grade.

### Predicted Detoxification Effect of Licorice on Cisplatin Hepatotoxicity

The chemical components of GC were obtained from the TCMSP database (Ru et al., 2014), in which drug-likeness (DL) ≥ 0.18 and oral bioavailability (OB) ≥ 30% were selected as potential active components (Yao et al., 2013), and glycyrrhizin and glycyrrhetinic acid were added with the reported detoxifying components (Wu et al., 2015). In total, 94 compounds were selected as active compounds in GC, and STITCH and TCMIP (Mao et al., 2019a) were used to predict the potential targets of GC. 277 potential targets were predicted with a combined score larger than 0.4, and identified compounds and targets are listed in Supplementary Table S1. Then, the TOXNET database (Wexler, 2001) was used for the collection of CP predicted toxic targets. In total, 1,048 potential toxic targets were predicted with a combined score larger than 30. Moreover, 82 common targets were found to be both CP toxic targets and GC predicted targets.

Tissue localization was analyzed by the BioGPS gene annotation database (Wu et al., 2016), and tissue in which the mRNA expression level of the common targets was higher than the average was selected as a potential target organ for toxicity, and a “target-tissue” localization network was constructed. A common targets function network was constructed, consisting of GC compounds, CP, common targets, and interactions of the drug-target. Next, common targets were submitted to DAVID Bioinformatics Resources 6.8 software to employ bioinformatics analysis.

The 3D structures of active constituents of GC and critical common targets were collected. Then, the hydrogenation and atomic type definitions were completed by AutoDock tools 4.2, and the grid was used to set grid parameter of receptors as follows: Spacing (angstrom) to 1, exhaustiveness to 10,000, z-dimension value of number and the center value were set to contain the entire receptors with docking area, and the rest was set to default. The AutoDock Vina program was run to dock active constituents with critical common targets.

### Animal Study

Male Wistar rats, 9 weeks of age, were obtained from the specific-pathogen free (SPF) animal laboratory of Gansu University of Chinese Medicine (Gansu, China). All animal studies were approved by the Ethic Committee of Gansu University of Chinese Medicine (Gansu, China) with approval number 2019-049, and followed related regulations. Rats were randomly divided into six groups (*n* = 10 rats per group): 1) control, 2) CP, 3) CP + GC at 255 mg/kg daily (intragastric administration, ig), 4) CP + GC at 450 mg/kg daily (ig), 5) CP + GC at 900 mg/kg daily (ig), and 6) GC (900 mg/kg daily, ig), and were continually fed for 8 days. On day 5, after 30 min of administration, except for the control and GC groups, rats were given an intraperitoneal injection of CP (8 mg/kg) to induce liver injury (Neamatallah et al., 2018). After 24 h of fasting at the last administration of GC, rats were anesthetized with sodium pentobarbital, blood was collected from the abdominal aorta, and serum and plasma were prepared and frozen at −20°C for future analysis. Subsequently, rats were euthanized, blood in liver tissues was removed by perfusion with saline, and the liver index was calculated (Qi et al., 2017), frozen at −80°C. Alanine aminotransferase (ALT) and aspartate aminotransferase (AST) levels in serum were determined by an automatic biochemical analyzer (Beckman Coulter, Shanghai, China) (Xiang et al., 2018).

### Histopathology and Immunohistochemistry

Liver tissues were stained with hematoxylin and eosin (H&E). Its morphology was examined by pathologists and imaged by light microscopy (DMI6000 B microscope, Leica, Wetzlar, Germany). The degree of liver damage was evaluated by a semiquantitative Knodell liver score, independently scoring by four indicators of inflammation, including necrosis, fibrosis, inflammatory volume, and structural change as follows: 1) the degree of necrosis around the manifold: 0–10 points; 2) the degree of lobular degeneration and focal necrosis: 0–4 points; 3) the inflammatory volume in the portal area: 0–4 points; and 4) the degree of liver fibrosis: 0–4 points. 10 visual fields were randomly selected from each section (Knodell et al., 1981).

5-μm thick deparaffinized tissue sections were hydrated and incubated overnight with rabbit anti-HMGB1, anti-p53, and anti-caspase-3 antibodies at 4°C, washed, and incubated with the secondary antibody. After washing, chromogen liquid DAB was applied, and hematoxylin staining was conducted. And its histopathological changes were observed using a light microscope (DMI6000 B microscope, Leica, Wetzlar, Germany) and analyzed by Image Pro Plus (Meng et al., 2018).

### TUNEL Assay

Deparaffinized liver tissue sections were hydrated, digested with proteinase K, and the apoptosis of liver was detected via an *in situ* apoptosis detection kit (Roche Applied Science, Mannheim, Germany) (Shunmugavel et al., 2015; Qi et al., 2017).

### Measurement of Superoxide Dismutase, Malondialdehyde, B-Cell Lymphoma/Leukemia 2, Tumor Necrosis Factor Alpha, and Interleukin 1β Levels

A total of 0.1 g liver tissue was added to 0.9 ml physiological saline, a homogenate was prepared, the supernatant was collected, and its protein concentration was determined using a rat total protein assay kit (Jiancheng, Nanjing, China). Subsequently, rat SOD activity assay kit (Solarbio, Beijing, China), rat MDA content assay kits (Solarbio, Beijing, China), rat Bcl-2 ELISA kit (Elabscience, Wuhan, China), rat TNF-α ELISA kit (Elabscience, Wuhan, China), and rat IL-1β ELISA kit (Cusabio, Wuhan, China) were used. All kits were performed according to the manufacturer’s guidelines. Each kit consisted a 96-well plate into which a specific antibody against a target protein, and wells were incubated with a horseradish peroxidase (HRP)-conjugated secondary antibody for colorimetric quantification (Wei et al., 2019a). The OD was read on a microplate reader at 450 and 560 nm. For each sample, experiments were performed in triplicate. Results were analyzed by *t*-text and *p* < 0.05 was considered significant.

### Protein Sample Preparation/Protein Trypsin Digestion

Liver tissues were homogenized in PBS with a tissue homogenizer (Sceintz-48, Sceintz, Ningbo, China), and each group contained liver tissues from three rats. A total of 0.1 g pooled tissues was lyzed with 400 μL urea lysis buffer (8 M Urea, 100 mM Tris-HCl pH 8.0), and 4 μL protease inhibitor (Pierce™, Thermo Fisher Scientific) was added (Wang et al., 2018). Protein concentrations were determined using the Bradford method (Eppendorf BioSpectrometer).

A total of 100 μg liver proteins were digested by the FASP procedure. Protein samples were added to 5 mM final concentration by 1 M dithiothreitol and were incubated for 30 min at 132.8°F, then protein samples were added to 20 mM final concentration by iodoacetamide and were incubated in the dark at 77°F for 30 min, and to a 5 mM final concentration by adding DTT in the dark at 15 min. Protein samples were loaded into 10 kD Microcon filtration devices (Millipore) and centrifuged, then, were loaded into 10 kD Microcon filtration devices (Millipore) and centrifuged, washed twice, respectively, with Urea lysis buffer (8 M urea, 100 mM Tris-HCl pH 8.0), and 50 mM NH_4_HCO_3_, and digested overnight at 98.6°F by trypsin (an enzyme to protein mass ratio of 1:25). Then, peptides were extracted and dried in a SpeedVac (Eppendorf).

### LC-ESI-MS/MS Measurements and Protein Quantification

Liquid chromatography (LC) (Bruker nanoElute) was coupled online to a hybrid TIMS quadrupole time-of-flight mass spectrometer (Bruker timsTOF Pro) via a CaptiveSpray nanoelectrospray ion source. LC was performed at 140°F and with a constant flow of 600 nL/min using a reversed-phase column (25 cm, 150 μm i.d.) with a pulled emitter tip, packed with 1.9 μm C18-coated porous silica beads (Dr. Maisch, Ammerbuch-Entringen, Germany) (Meier et al., 2018). Mobile phases A and B were water with 0.1% formic acid (v/v) and ACN with 0.1% formic acid (v/v), respectively. Peptides were separated linearly gradient from 3% to 15% B within 30 min, 24% B within 40 min, 35% within 30 min, and by a washing step at 95% B and re-equilibration. The dual TIMS analyzer was operated at a fixed duty cycle close to 100% via equal accumulation and ramp times of 130 ms each. Data-dependent data acquisition was performed in PASEF mode with five PASEF scans per topN acquisition cycle. Singly charged precursors were excluded by their position in the m/z-ion mobility plane, and precursors that reached a “target value” of 5,000 a.u. were dynamically excluded for 18 s. The quadrupole isolation width was set to two Th for m/z < 700 and three Th for m/z > 700 (Wang et al., 2018).

Raw MS files were managed by MaxQuant software (version 1.6.6.0), and its Andromeda search engine was used to identified MS/MS-based peptide, which uses a target-decoy approach to identify proteins at an FDR <1%. The rat ref-sequence protein database (updated at 2019/05/20, 66858 proteins ID in total) was used as a forward database and the reverse database of decoy search was autogenerated by MaxQuant. “Trypsin” was set for enzyme specificity, and at least seven amino acids were required to identify peptides. Default settings were used for variable and fixed modifications (variable modification: acetylation (protein-N terminus) and oxidation methionine (M)), fixed modification: carbamidomethylation (C). A MaxQuant label-free quantification (LFQ) algorithm was used to quantitate the MS signals, and the intensities of proteins were represented in intensity based absolute protein quantification (iBAQ) (Wei et al., 2019b). The iBAQ of each sample was transferred into a fraction of total protein iBAQ amount per experiment (FOT).

The mass spectrometry proteomics data have been deposited to the ProteomeXchange Consortium via the PRIDE (Deutsch et al., 2020) partner repository with the dataset identifier PXD021688.

### Bioinformatics Analysis

The iBAQ value was used as the protein expression, and an average value of three samples in each group was taken. The variation trend of the expression of the same protein in each group was analyzed. The cellular component, molecular function, and biological processes were analyzed by DAVID Bioinformatics Resources 6.8 software.

### Western Blot Analysis

Proteins from liver samples were extracted by precooling RIPA lysis buffer (Solarbio, China), loaded on 10% SDS-PAGE gels, and proteins were transferred to PVDF membranes (Milipore, United States). Activated membranes were transferred in protein blots, blocked in 5% nonfat milk within 1.5 h, and then incubated overnight at 4°C with one of the following primary antibodies: anti-GAPDH (1:2,000), anti-cytochrome P450 4A1/2 (CYP4A2, 1:1,000), anti-CYP1A1 (1:100), anti-CYP1A2 (1:500), anti-Abcb1b (1:1,000), anti-ABCC2 (1:500), anti-ESR1 (1:1,000), and anti-TOP2A (1:5,000). Then, membranes were washed with Tris-buffered saline containing Tween 20 (TBST) and incubated at 24°C for 1 h with a second antibody, and visualized by an enhanced chemiluminescence kit (Millipore, United States). Results were analyzed by *t*-test, and *p* < 0.05 was considered significant.

## Results

### Prediction of Detoxification of Licorice on Cisplatin

**FIGURE 1:**
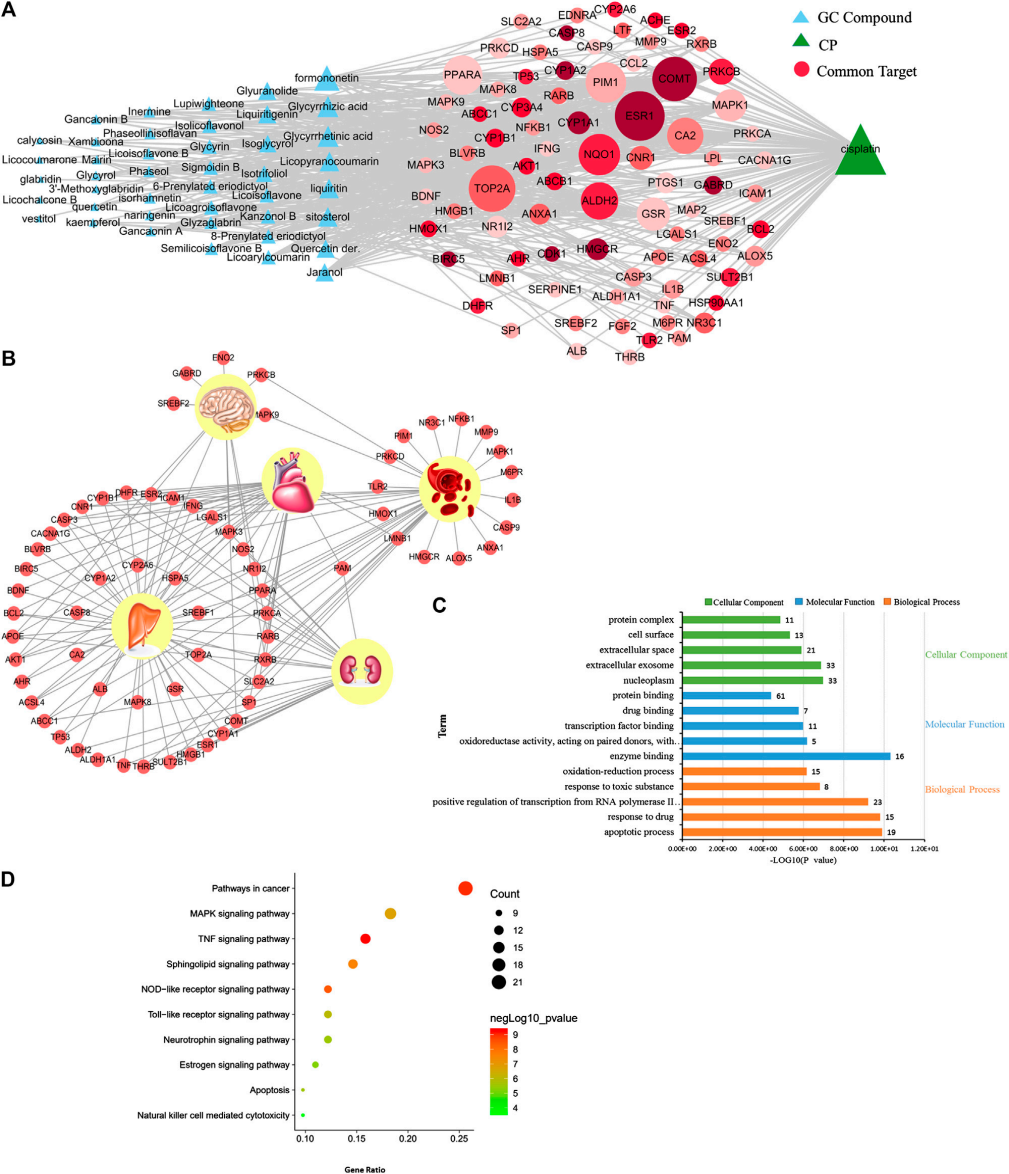
Prediction results of network pharmacology of detoxification in licorice on cisplatin. **(A)** Network of licorice (GC) compounds, cisplatin (CP), and common targets. **(B)** Network of “common target-tissue” localization. **(C)** Cellular components, molecular functions, and biological processes associated with common targets of detoxification in GC on CP. **(D)** Pathways associated with common targets of detoxification in GC on CP.

The relationships between GC and the toxicity of CP were investigated by network analysis as shown in Figure 1A. In the network, the size of node was positively correlated with its degree, and common targets were sequenced with color depth by combined scores in GC. The “common target-tissue” network (Figure 1B) was built to investigate the toxicity tissue, induced by CP, which common targets were located in. A total of 45 out of 82 common targets were located in the liver, and multiple targets were located in the liver, kidney, and heart.

Next, bioinformatics analysis of common targets was conducted (Figures 1C,D). The detoxification of GC on CP is most likely associated with the TNF signaling pathway, NOD-like receptor signaling pathway, MAPK signaling pathway, apoptosis, neurotrophin signaling pathway, estrogen signaling pathway, and so on. Thus, our data suggest that GC may regulate multiple targets by a variety of compounds to have a detoxification effect on CP-induced hepatotoxicity.

**FIGURE 2:**
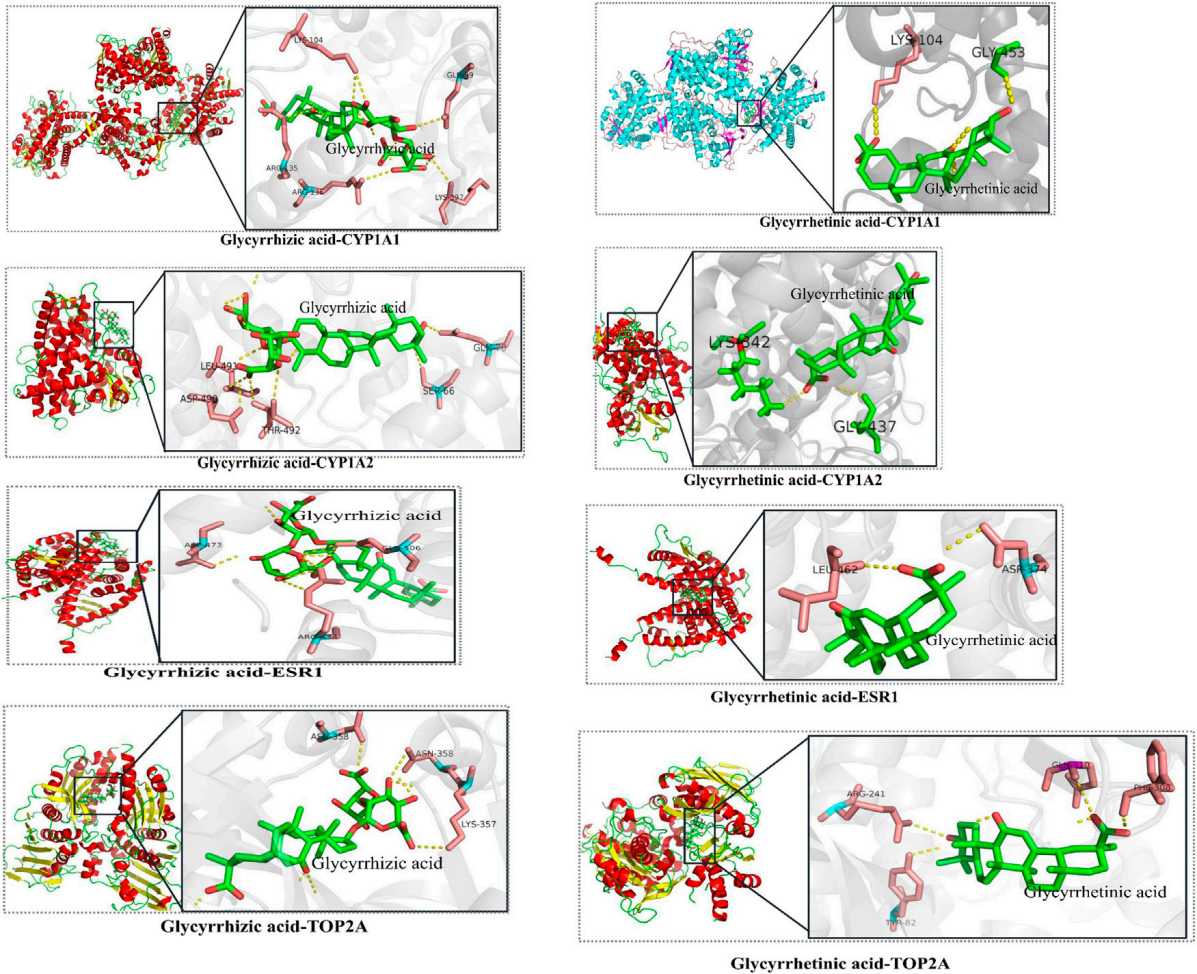
The results of molecular docking of candidate critical licorice (GC) components and critical common targets.

### Verification of the Predicted Detoxification of Licorice on Cisplatin Hepatotoxicity

The candidate critical components of GC were docked with the critical common targets to verify the accuracy of the prediction mentioned above. Glycyrrhizic acid and glycyrrhetinic acid were successfully docked with CYP1A1, CYP1A2, ESR1, and TOP2A (Figure 2, details are listed in Supplementary Table S1), which suggested that GC may play a synergistic role in detoxification by multiple compounds and targets.

### Licorice Attenuated Cisplatin-Induced Liver Injury in Rat Models

**FIGURE 3:**
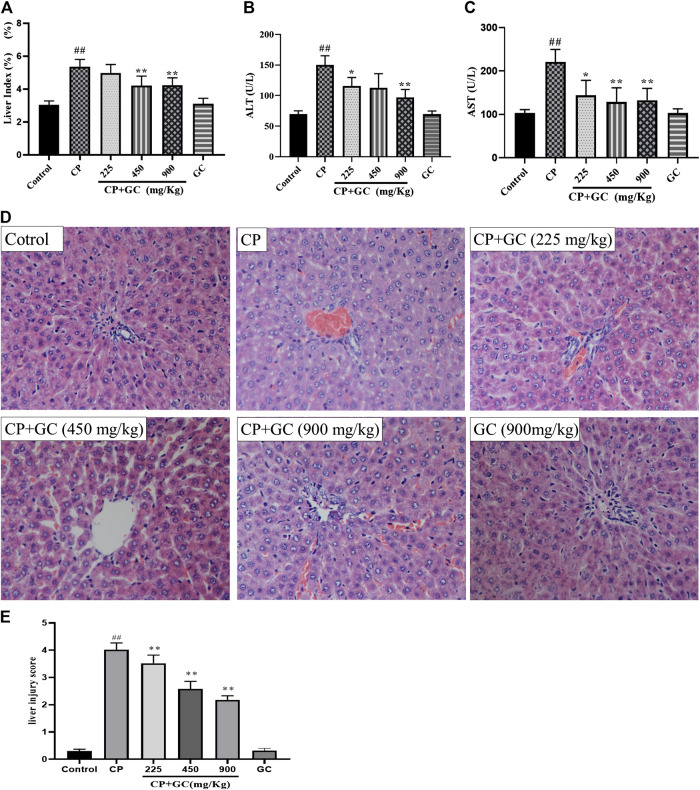
Detoxification of licorice (GC) on cisplatin (CP)-induced hepatotoxicity in rats. **(A)** Liver index in rats (*n* = 10). **(B)** Serum aspartate aminotransferase (AST) levels (*n* = 10). **(C)** Serum alanine aminotransferase (ALT) levels (*n* = 10). **(D)** HE histological staining of liver tissue (200×). **(E)** Liver histological score of histological staining (*n* = 10). The results are presented as the mean ± SD. Significant differences vs. those in the control group (^##^ *p* < 0.01) and those of the CP-treated group (^*^ *p* < 0.05; ^**^ *p* < 0.01) were determined by *t*-test.

The liver index and serum levels of ALT and AST were used to assess the detoxification of GC on CP-induced hepatotoxicity. After CP administration, the liver index and the serum levels of ALT and AST were significantly increased when compared with the control (*p* < 0.01; Figures 3A–C), whereas they were reduced by GC. Moreover, the above-mentioned indicators were not significantly different when using high doses of GC without CP from the control, which suggested that the experimental dose of GC did not induce hepatotoxicity in rats.

Histological staining of liver (Figures 3D,E) showed that the hepatocyte in rats in control had intact cytoplasm, nucleus, and nucleolus (green arrow), and the central vein (blue arrow) was visible. Liver injury was induced by CP, which was manifested as a change in the structure of hepatic lobules, karyopyknosis (black arrow), hepatocyte swelling, ballooning degeneration (red arrow), local or scattered bleeding (yellow arrow), and a high liver histological score. Moreover, GC remarkably improved the liver injury and significantly reduced the liver histological score (*p* < 0.05) when compared with CP. Taken together, these results showed that GC alleviated liver injury to reduce CP-induced hepatotoxicity in rats. Moreover, hepatic pathological changes mentioned above were not found in GC administered alone group, which means liver injury was not induced by GC in 900 mg/kg.

### Effects of Licorice on Indicators Related to Apoptosis, Oxidative Stress, and Inflammation in Cisplatin-Induced Hepatotoxic Rats

**FIGURE 4:**
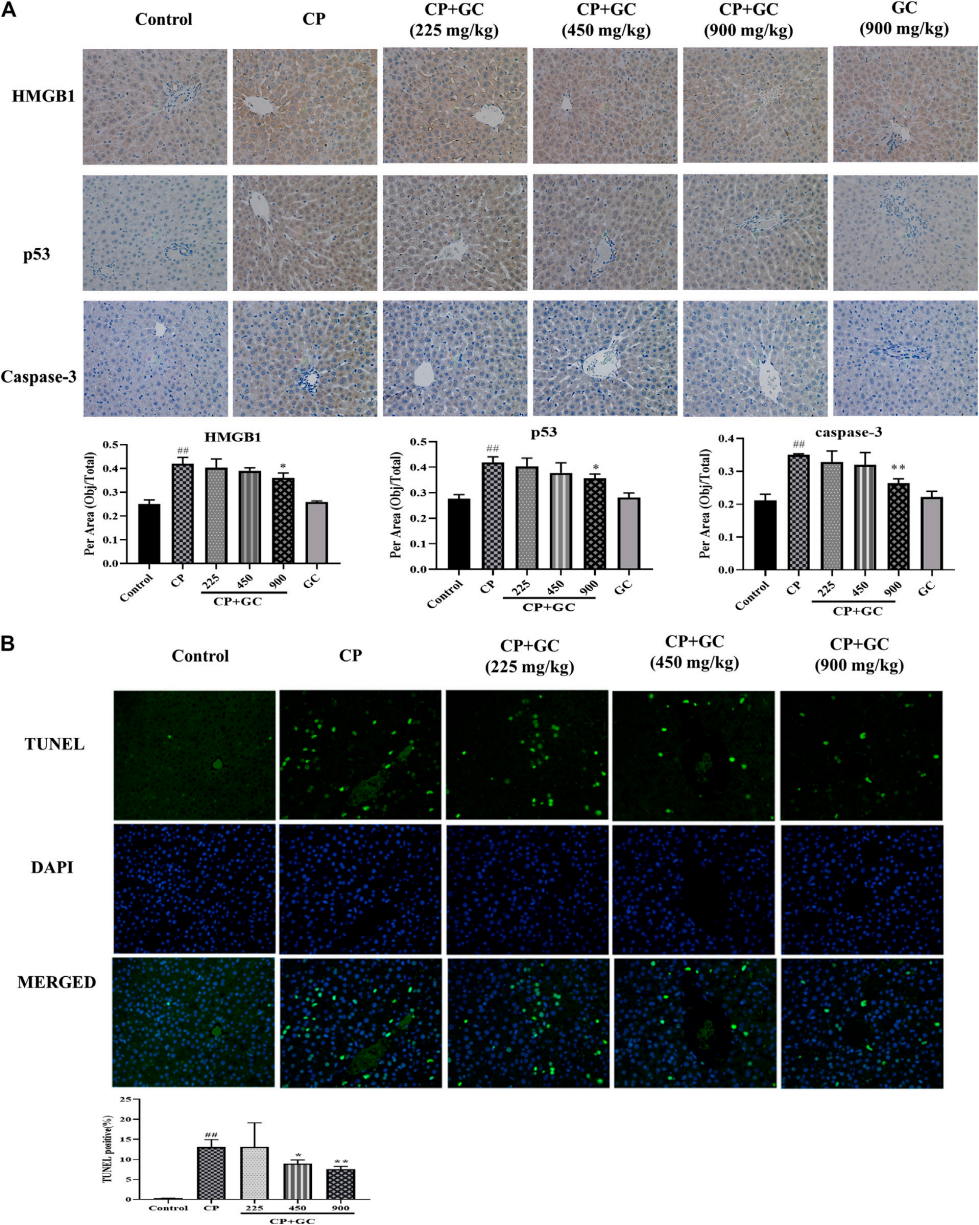
The results of immunohistochemical and TUNEL staining. **(A)** Immune expression of HMGB1, p53, and caspase-3 in liver tissue (*n* = 3). **(B)** Results of TUNEL staining in rat livers (*n* = 3). Results are presented as the mean ± SD. Significant differences vs. those in the control group (^##^ *p* < 0.01), and those of the CP-treated group (^*^ *p* < 0.05; ^**^ *p* < 0.01) were determined by *t*-test.

**FIGURE 5:**
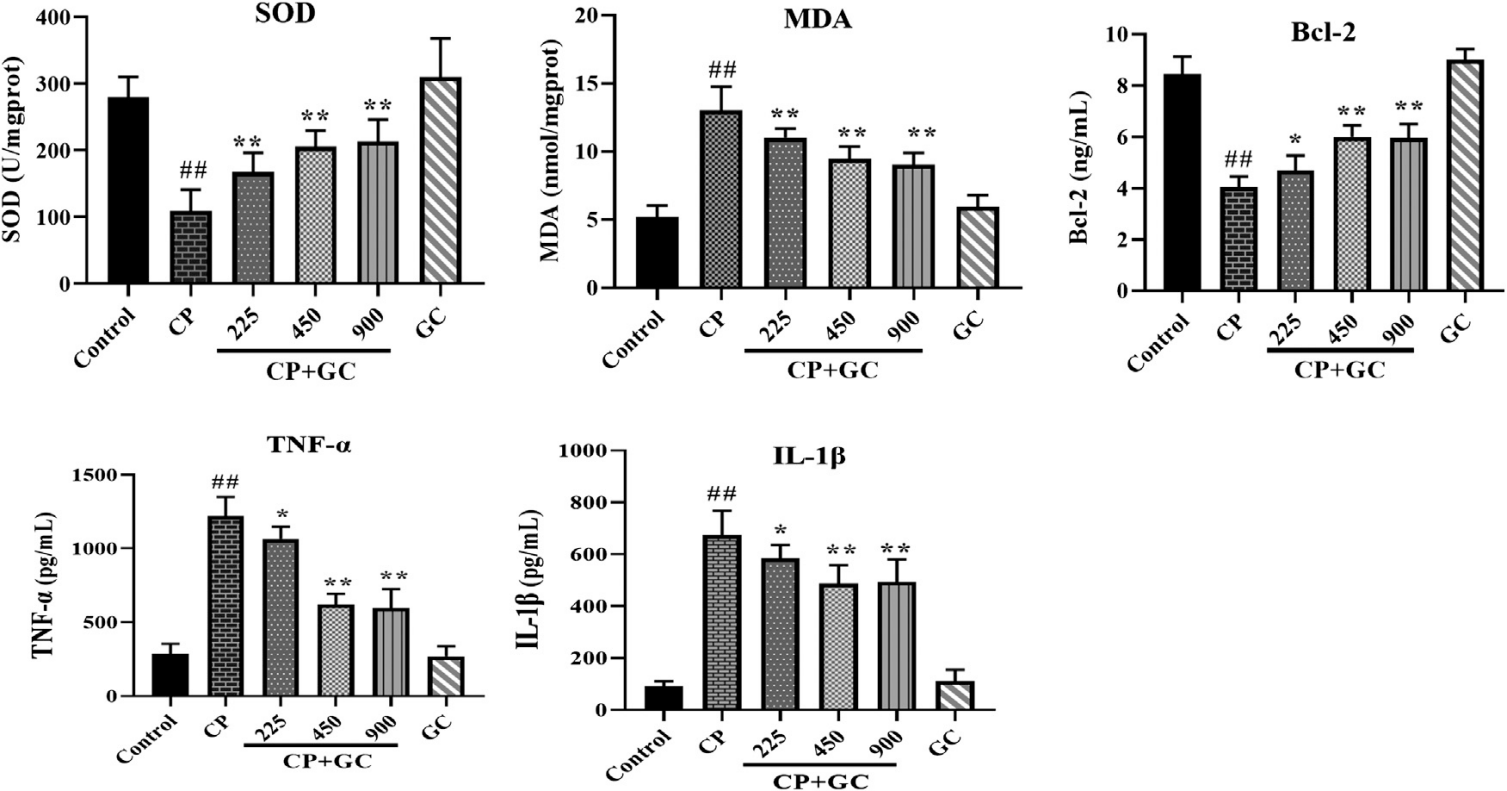
Effects of licorice on the serum levels of superoxide dismutase (SOD), malondialdehyde (MDA), Bcl-2, TNF-α, and IL-1β on cisplatin (CP)-induced hepatotoxicity in rats (*n* = 10). The results are presented as the mean ± SD. Significant differences vs. those in the control group (^##^ *p* < 0.01) and those of the CP-treated group (^*^ *p* < 0.05; ^**^ *p* < 0.01) were determined by *t*-test.

The predicted detoxification of GC on CP is most likely associated with apoptosis. Therefore, the protein expression of caspase-3, p53, and Bcl-2 was determined by immunohistochemistry or ELISA. The positive expression of p53 located in the nucleus and that of caspase-3 in the cytoplasm was rare in liver tissue of control rats (Figure 4A), and the positive expression rates of p53 and caspase-3 were higher in CP treated rats (*p* < 0.01). The positive expression after GC pretreatment (900 mg/kg) was significantly decreased when compared to CP (*p* < 0.05). The ELISA results showed that levels of Bcl-2 in the liver were significantly decreased by CP (*p* < 0.01), and were effectively reversed after GC administration (Figure 5). Moreover, the TUNEL assay staining (Figure 4B) revealed that GC treatment significantly reduced the number of TUNEL-positive cells against CP-induced apoptosis (*p* < 0.05).

In addition, the TNF signaling pathway as well as the oxidation-reduction process was also predicted. Therefore, we evaluated the anti-inflammatory effect of GC in the liver on HMGB1, TNF-α, and IL-Iβ, and anti-oxidation on SOD and MDA. Upon liver injury, the expression of HMGB1 would be transferred from nucleus to cytoplasm (Zhou et al., 2020). The positive expression of HMGB1 in livers of control was mostly observed in the cell nucleus (Figure 4A), whereas transferred into the cytoplasm after CP treatment (*p* < 0.01), and the high expression in the cytoplasm was significantly reduced after GC-treatment (900 mg/kg). Moreover, in CP-induced injured livers, the levels of TNF-α, IL-Iβ, and MDA were increased and SOD levels were reduced when compared with the control (*p* < 0.01), whereas they were effectively reversed in a dose-dependent manner after GC administration (*p* < 0.05; Figure 5). Taken together, these results suggested that GC improved apoptosis, oxidative stress, and inflammation induced by CP against hepatotoxicity.

Furthermore, there was no significant difference neither in positive expression rates of HMGB1, p53, and caspase-3 or levels of SOD, MDA, Bcl-2, TNF-α, and IL-Iβ in GC administered alone group when compared with control (Figures 4, 5), which suggested again that the experimental dose of GC did not induce hepatotoxicity in rats.

### Proteomic Investigation of Detoxification of Licorice on Cisplatin Hepatotoxicity

**FIGURE 6:**
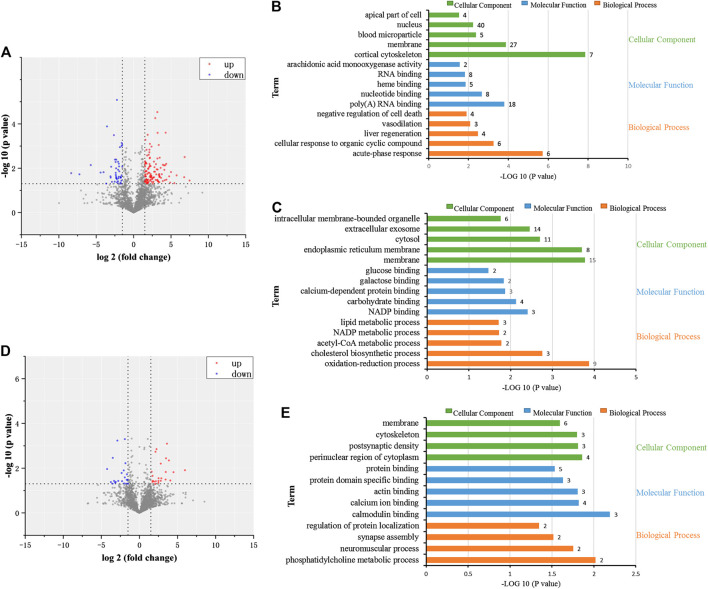
Proteomic investigation of the dynamic changes of proteins in detoxification of licorice on cisplatin (CP)-induced hepatotoxicity in rats. **(A)** Volcano plot of dynamic changes of proteins in CP-treated rats compared to control-treated rats. **(B)** Cellular constituents, biological processes, and molecular function of proteins in the CP group with upregulated expression levels (*p* < 0.05) when compared to the control. **(C)** Cellular constituents, biological processes, and molecular function of proteins in the CP group with downregulated expression levels (*p* < 0.05) when compared to the control. **(D)** Volcano plot of dynamic changes of proteins in GC-treated rats, compared to CP. **(E)** Cellular constituents and biological processes of the proteins of GC-treated rats with upregulated expression levels (*p* < 0.05) when compared to CP.

Liver tissues of rats in the control, CP, and CP + GC at 900 mg/kg were selected to investigate the dynamic changes in proteins by high-throughput proteomic profiling. A total of 4,248 proteins were identified (details are listed in Supplementary Table S2), and 158 proteins were more than 1.5-fold different in CP-treated rats when compared with control (*p* < 0.05). Among them, molecular function and biological processes of 111 upregulated proteins were related to acute-phase responses, cellular responses to organic cyclic compounds, liver regeneration, vasodilation, and negative regulation of cell death. In addition, 47 downregulated proteins were related to oxidation-reduction processes, cholesterol biosynthetic processes, acetyl-CoA metabolic processes, NADP metabolic processes, and lipid metabolic processes (Figures 6A–C). Furthermore, 36 proteins were more than 1.5-fold different in GC-treated rats compared with CP-treated rats (*p* < 0.05). Among them, 18 upregulated proteins were related to phosphatidylcholine metabolic processes, neuromuscular processes, regulation of protein localization, calmodulin binding, and calcium ion binding (Figures 6D,E). Furthermore, for a total of 18 downregulated proteins, no functional enrichment results were observed.

**FIGURE 7:**
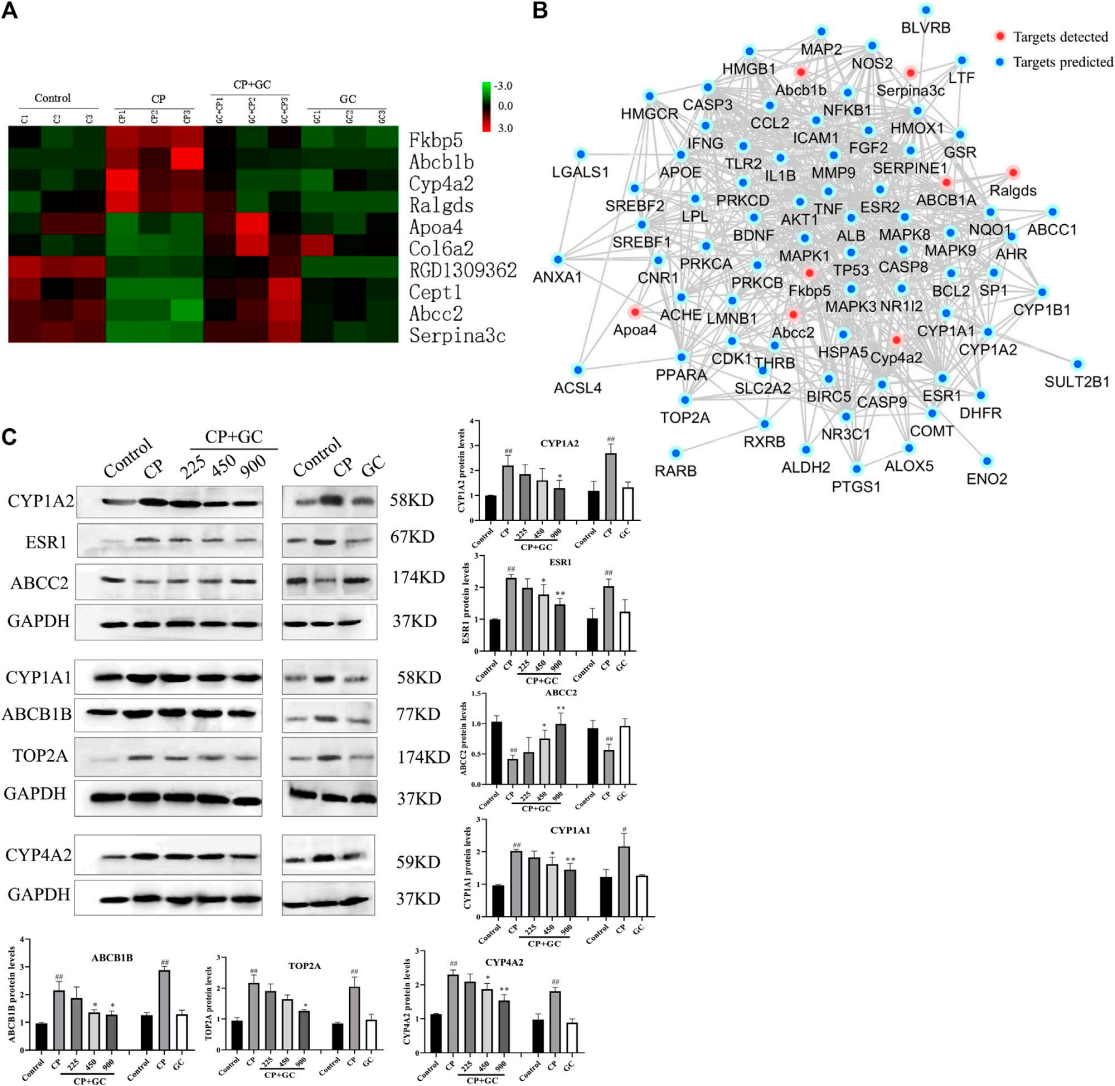
Proteomic investigation of critical proteins, the relation with predicted targets, and verification. **(A)** Dynamic protein changes and hierarchical clustering of alternative critical proteins in control, cisplatin (CP), licorice (GC)-treated, and GC administered alone rats (*n* = 3). Z-score transformation was used. **(B)** Network of predicted targets, detected proteins, and interactions between them. **(C)** Verification of critical proteins in detoxification of GC (*n* = 3). The results are presented as the mean ± SD. Significant differences vs. those in the control group (^##^ *p* < 0.01), and those of the CP-treated group (^*^ *p* < 0.05; ^**^ *p* < 0.01) are determined by *t*-test.

Subsequently, 10 proteins that showed over a 1.5-fold difference between groups, which showed the same trend in CP-treated rats vs. control-treated rats and GC-treated rats vs. CP-treated rats, were considered candidate critical proteins for the detoxification of GC (Figure 7A). However, for predicted common targets, only ABCB1 was identified, which indicated that the molecular mechanisms of detoxification of GC were rather complex. Therefore, a network was constructed to investigate the interaction between differential proteins detected by proteomics and critical targets predicted (Figure 7B). The network contained eight differential proteins, 73 predicted targets, and interactions among these effectors. According to the fold changes, network, and literature, the detected differential proteins (CYP4A2, ABCB1B, and ABCC2), and the predicted critical targets (CYP1A1, CYP1A2, ESR1, and TOP2A) were selected for further verification.

**FIGURE 8:**
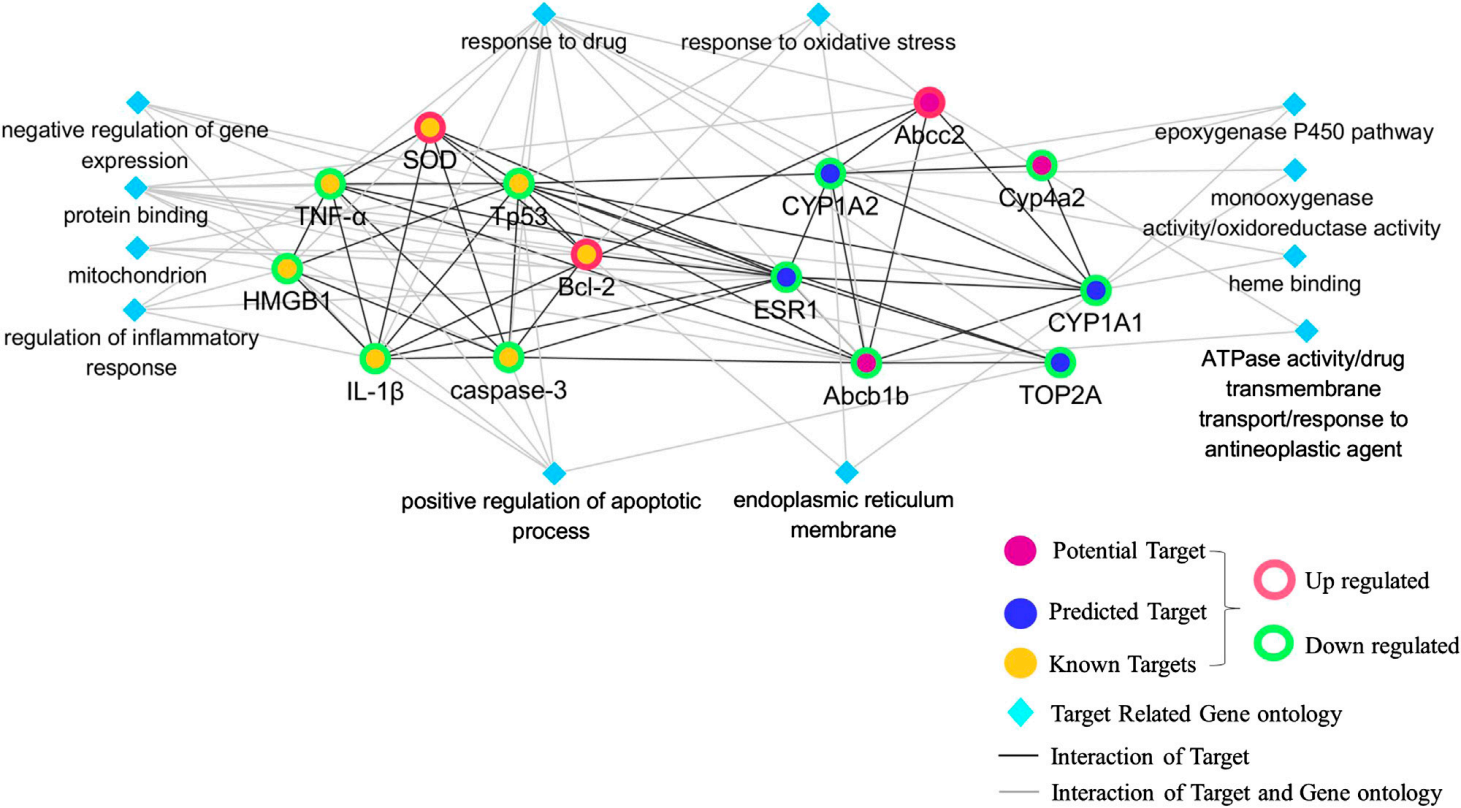
Network of critical proteins, known targets, and molecular functions or biological processes among them.

### Verification of Critical Proteins in Detoxification of Licorice to Cisplatin Hepatotoxicity

The expression levels of candidate critical proteins were determined by Western blot analysis. Figure 7C shows that when compared with the control, the expression levels of CYP1A2, CYP1A1, ESR1, TOP2A, Cyp4a2, and ABCB1B in CP-induced injured liver tissues were significantly increased (*p* < 0.05) and ABCC2 levels were significantly decreased (*p* < 0.05), whereas they were effectively reversed by GC pretreatment in a dose-dependent manner. Among them, the expression levels of Cyp4a2, ABCB1B, and ABCC2 were consistent with the data obtained by proteomics. Moreover, there was no significant difference in the expression levels of candidate critical proteins in GC administered alone group when compared with control.

### Network of Critical Proteins, Known Targets, and Their Interaction

A network of critical proteins, known targets and cellular components, molecular functions, and biological processes was constructed to investigate the molecular mechanism of detoxification of GC against CP-induced hepatotoxicity (Figure 8). Verified critical proteins, ABCB1B, ABCC2, CYP4A2, CYP1A1, CYP1A2, ESR1, and TOP2A were selected. Our data showed that the critical proteins have direct interactions with each other, and with known targets measured in the model above, including SOD, HMGB1, caspase 3, p53, Bcl-2, TNF-α, and IL-1β. In addition, all these proteins played a detoxifying role of GC on CP-induced hepatotoxicity by correlating with oxidoreductase activity, response to oxidative stress, drug transmembrane transport, regulation of inflammatory responses, positive regulation of apoptotic processes, the epoxygenase P450 pathway, and responses to drugs.

## Discussion

Currently, CP is one of the most effective antitumor drugs, but has serious adverse reactions, including nephrotoxicity, ototoxicity, hepatotoxicity, and genetic toxicity (Tsang et al., 2009; Macciò and Madeddu, 2013). GC, a traditional herbal medicine, is commonly used to detoxicate poisonings from poisons and drugs, and has a hepatoprotective activity on drug-induced liver injury (Kuang et al., 2017; Wu et al., 2020), which indicated that GC may be an auxiliary drug to CP with hepatotoxicity-reducing effects. However, further studies will need to be performed to verify these findings. Thus, we observed the possibility of detoxification and revealed the molecular mechanism of GC to CP by establishing a CP-induced hepatotoxicity model in rats.

In this study, we confirmed that GC can recover functional indices and liver pathological injury in the injured liver to exert favorable effects against CP-induced hepatotoxicity. However, as a Traditional Chinese Medicine (TCM), it is a challenge for GC to determine the effector molecules involved, reveal the underlying molecular mechanism of action, and locate its detoxification to CP-induced hepatotoxicity in the clinic. In our study, a systematic approach based on network pharmacology and proteomics was established to provide a panoramic and accuracy view of the comprehensive efficacy of GC by predicting the detoxification, investigating the detoxification of GC in rats, and by analyzing the changes in protein expression in the liver.

Apoptosis, oxidative stress, and inflammatory responses were evident after treatment with CP (Rehman et al., 2014), which was also confirmed by our results of network pharmacology. p53 can be activated in the liver and can regulate the transcription of downstream genes, including Bcl-2 and Bax (Mao et al., 2019b). Bcl-2, an antiapoptotic factor, can inhibit apoptosis and promote cell growth by binding to the proapoptotic factor Bax (Willis et al., 2003). As a biomarker of irreversible apoptosis, caspase-3 has been shown to participate in the apoptotic cascade (Marshall et al., 2018). HMGB1, a DNA-binding protein, is closely related to a variety of pathophysiological processes, which can increase local inflammatory responses, leading to apoptosis or necrosis (Pang et al., 2016). Furthermore, SOD reflects the endogenous antioxidant capacity and protects cells from oxidative stress by scavenging oxygen free radicals (Poprac et al., 2017). MDA, an important indicator of oxygen radical damage, reflects the degree of lipid peroxidation (Ayala et al., 2014). Moreover, inflammasome such as NLRP3 could be activated by oxidative stress to further induce liver inflammatory and hepatocyte apoptosis (Abbasi-Oshaghi et al., 2019). In our study, the downregulation of p53 and caspase-3, upregulation of Bcl-2, and results obtained by the TUNEL assay in liver tissues by GC indicated that GC inhibited hepatocyte apoptosis against hepatotoxicity. Moreover, the SOD activity was elevated, whereas the levels of MDA, HMGB1, TNF-α, and IL-1β were decreased in liver injury in rats, thereby suggesting that the detoxification of GC on CP hepatotoxicity was related to against oxidative stress and inflammatory responses.

**FIGURE 9:**
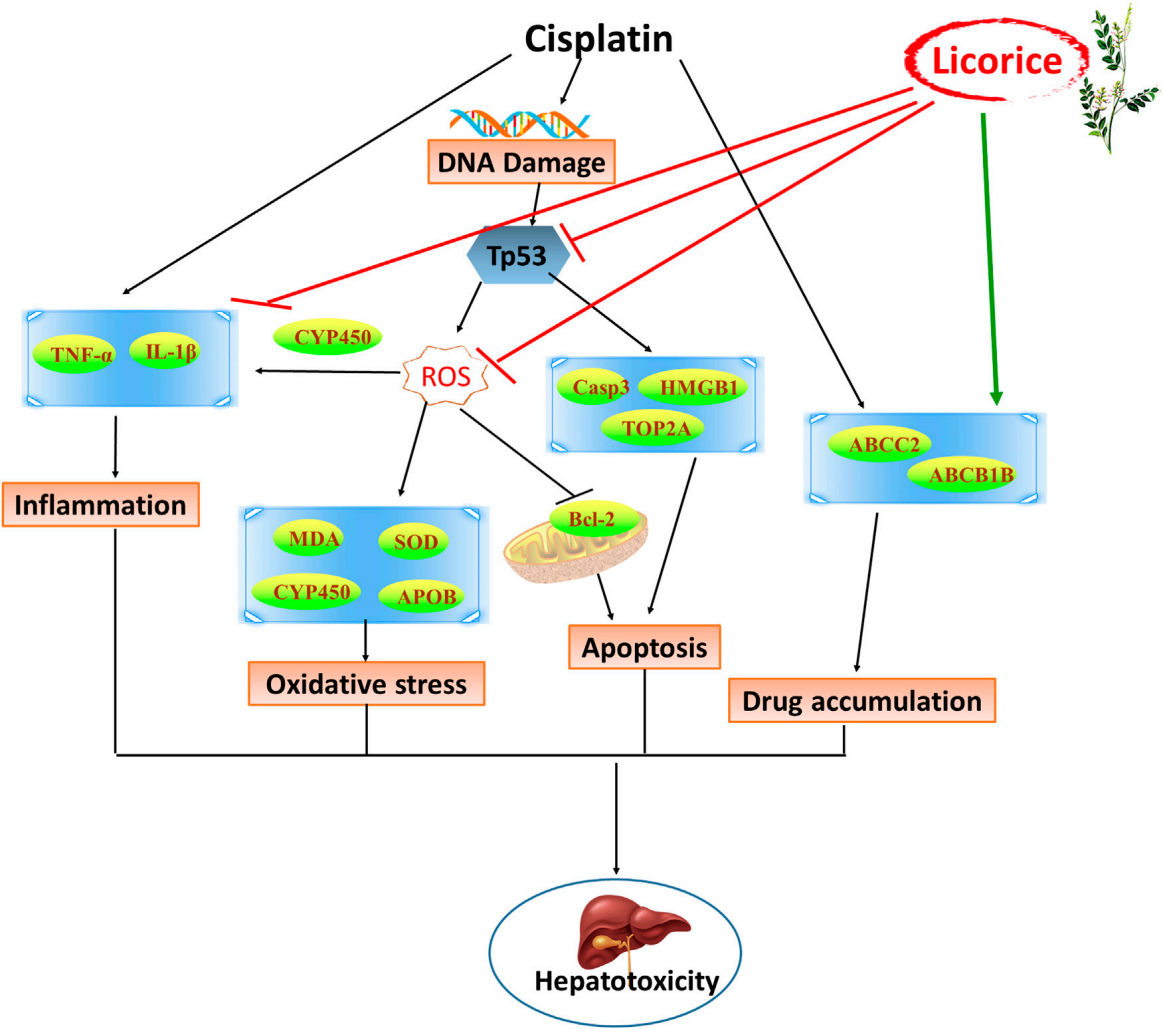
Schematic diagram of the detoxification of licorice on cisplatin-induced hepatotoxicity.

In this study, our findings indicated that the unusual expression levels of CYP1A1, CYP1A2, CYP4A2, ABCB1B, ABCC2, ESR1, and TOP2A on CP-induced hepatotoxicity in rats can be recovered by GC. It has been reported that the mechanisms of detoxification of GC were related to the regulation of P450, reducing toxic activation, inducing metabolism, interfering with P-glycoprotein (P-gp) expression, and accelerating excretion (Wang et al., 2013). Cytochrome P450 enzyme (P450) is the most common metabolic enzyme in phase I metabolism, which can change the molecular structure of drugs, increase the polarity of drugs, and cause drug detoxification or toxicity reactions (Foroozesh et al., 2019). CYP1A1 and CYP1A2 were involved in toxic metabolism (Go et al., 2015), and inhibition of CYP1A1 and CYP1A2 protected the liver from drug-induced DNA damage (Cui et al., 2017). CYP4A2 can induce liver toxicity by proliferative responses of peroxisomes and oxidative stress (El-Sherbeni et al., 2013), whereas CCl_4_-induced liver injury could be improved by decreasing the expression of CYP4A2 (Yang et al., 2014). ABCB1 can encode P-gp and plays a key role in drug uptake, distribution, metabolism, and excretion (Marchetti et al., 2007). P-gp has been reported to regulate resistance and toxicity induced by CP (Ding et al., 2017), and plays an important detoxifying role of GC to other drugs (He et al., 2019). ABCC2 encodes multidrug resistance-associated protein 2 (MRP2) to involve in drug transport and excretion (Baiceanu et al., 2015), and drug-toxicity in a patients’ livers could be enhanced by inducing its transport activity (Pedersen et al., 2008). And the glycyrrhizin, an active constituent of GC, had hepatic protection by inhibiting ABCC2-encoded MRP2 (Zhou et al., 2016). Moreover, ABCB1 and ABCC2 could play an overlapping function in elimination of hepatotoxicity of antitumor drugs (Lagas et al., 2010), and in drug resistance of tumor cells to CP (Wang et al., 2017). As a key transcription factor of CYP3A4, ESR1 regulated the expression of CYP3A4 and other P450, thereby having broad effect on the metabolism of xenobiotics in human liver (Wang et al., 2019). ESR1 could mediate aggravation of chemical-induced liver injury and inhibition of liver regeneration (McGreal et al., 2017). TOP2A was closely related to the proliferation of various cancer cells (Chen et al., 2017; Liu et al., 2018). Overexpression of TOP2A increased hepatocyte viability and participated in drug-induced hepatotoxicity (Chen et al., 2018), and is an important biomarker involved in hepatocyte proliferation during liver regeneration in rats (Bai et al., 2019). The expression of ABCB1B, CYP4A2, CYP1A1, CYP1A2, ESR1, TOP2A, and ABCC2 in CP-induced hepatotoxicity rats can be reversed by GC pretreatment in a dose-dependent manner, which is crucial in the transmembrane transport of drugs and CP hepatotoxicity processes. However, only few predicted targets could be identified due to limited coverage of proteomics and having low-abundance proteins or wrong predicted targets probably, which indicated that the molecular mechanism of the multitarget effect from GC to CP-induced hepatotoxicity is complex. Fortunately, some proteins were indeed identified that were closely related to oxidative stress, apoptosis, and inflammation-related processes induced by CP, and were consistent with the efficacy of GC (Figure 9).

In conclusion, GC plays a detoxifying role through multitargets and multifunction, and can be used as an adjuvant drug candidate for CP to effectively reduce hepatotoxicity by inhibiting oxidative stress, apoptosis, inflammatory responses, and accelerating metabolism.

## Data Availability Statement

The original contributions presented in the study are publicly available. These data can be found here: http://www.ebi.ac.uk/pride with the accession number: PXD021688.

## Ethics Statement

The animal study was reviewed and approved by the Animal Care Welfare Committee of Gansu University of Chinese Medicine (Gansu, China, 2019-049) and followed related regulations.

## Author Contributions

QM conceived the study, conducted the proteomics, and wrote the manuscript. YD conceived the study. PL, JM, and XY performed the animal study. ZY, YZ, and XJY conducted ELISA and Western blot experiments. All authors reviewed and approved the final manuscript.

## Funding

This work was supported by National Natural Science Foundation of China (No. 81960723), Lanzhou science and the technology planning project (No. 2018‐4‐63), Open Foundation project of Key Laboratory of Pharmacology and Toxicology of Chinese Medicine in Gansu Province (No. ZDSYS‐KJ‐2018-09), and Foundation of Chengdu Medical college (No. CYZZD 20‐02).

## Conflict of Interest

The authors declare that the research was conducted in the absence of any commercial or financial relationships that could be construed as a potential conflict of interest.
